# Research Progress on the Mechanism of Nanoparticles Crossing the Intestinal Epithelial Cell Membrane

**DOI:** 10.3390/pharmaceutics15071816

**Published:** 2023-06-25

**Authors:** Yunjie He, Meng Cheng, Ruyue Yang, Haocheng Li, Zhiyang Lu, Yi Jin, Jianfang Feng, Liangxing Tu

**Affiliations:** 1 National Pharmaceutical Engineering Center for Solid Preparation in Chinese Herbal Medicine, Jiangxi University of Chinese Medicine, Nanchang 330006, China; hbwhbw12345@163.com (Y.H.); jinyizju@hotmail.com (Y.J.); fengjianfang@vip.163.com (J.F.); 2 The Affiliated Hospital of Jiangxi University of Chinese Medicine, Nanchang 330006, China; cmjxzyy@126.com; 3 School of Pharmacy, Guangxi University of Chinese Medicine, Nanning 530200, China

**Keywords:** nanoparticles, intestinal epithelial cells, cell uptake, intracellular transport, basolateral exocytosis

## Abstract

Improving the stability of drugs in the gastrointestinal tract and their penetration ability in the mucosal layer by implementing a nanoparticle delivery strategy is currently a research focus in the pharmaceutical field. However, for most drugs, nanoparticles failed in enhancing their oral absorption on a large scale (4 folds or above), which hinders their clinical application. Recently, several researchers have proved that the intestinal epithelial cell membrane crossing behaviors of nanoparticles deeply influenced their oral absorption, and relevant reviews were rare. In this paper, we systematically review the behaviors of nanoparticles in the intestinal epithelial cell membrane and mainly focus on their intracellular mechanism. The three key complex intracellular processes of nanoparticles are described: uptake by intestinal epithelial cells on the apical side, intracellular transport and basal side exocytosis. We believe that this review will help scientists understand the in vivo performance of nanoparticles in the intestinal epithelial cell membrane and assist in the design of novel strategies for further improving the bioavailability of nanoparticles.

## 1. Introduction

Nanoparticles are nanometer-sized drug formulations that are prepared by dissolving, dispersing, adsorbing or wrapping drugs in suitable carrier materials [1]. Their particle sizes are generally within the range of 10–100 nm, which can improve the physicochemical properties and enhance the solubility and permeability of drugs [2,3]. Compared with traditional oral administration, nanoparticle delivery systems have unique advantages [4]: (1) they improve the stability and solubility of the drug in the gastrointestinal tract [5,6,7]; (2) they promote the penetration and adhesion of the drug in the mucus layer of the gastrointestinal tract, which is beneficial for the drug to reach intestinal epithelial cells [8,9,10,11]; (3) the controllable release of the drug can be achieved to maintain a stable blood drug concentration [12,13]; (4) by carrying out the surface modification of the nanoparticles, the drug can be targeted for delivery and can be released at the treatment site [14,15]; (5) they can change the absorption mechanism of the gastrointestinal tract and promote drug absorption [16,17,18].

**Figure 1:**
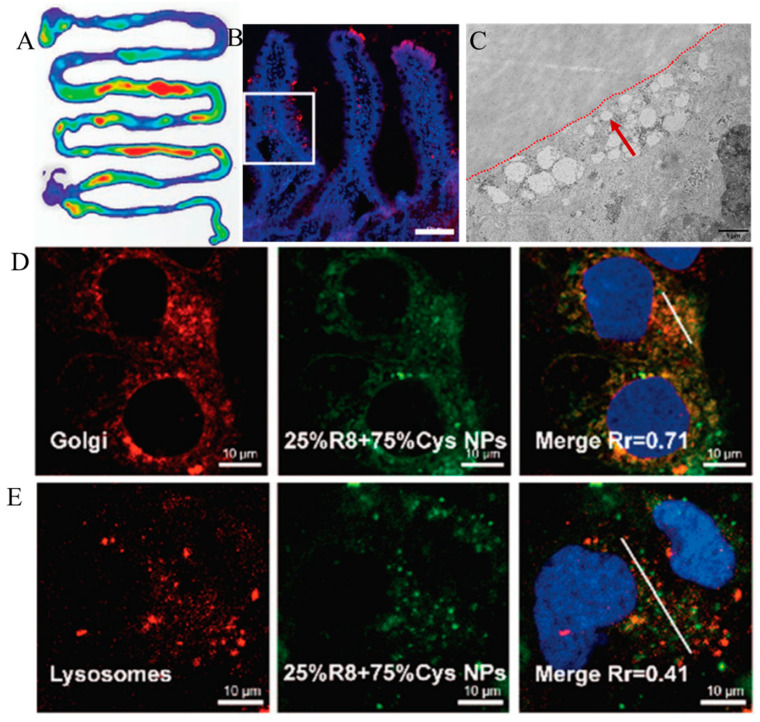
Schematic diagram of the intestinal absorption of nanoparticles. (**A**) Intestinal distribution of nanoparticles. Reprinted with permission from Ref. [24] (copyright 2021, Zhou). (**B**) Nanoparticle intestinal villus adsorption. Scale bar (CLSM): 100 μm. Reprinted with permission from Ref. [25] (copyright 2021, Xing). (**C**) Base-side exocytosis diagram of nanoparticles. Reprinted with permission from Ref. [26] (copyright 2021, Zhang). (**D**) Nanoparticle Golgi colocalization. Reprinted with permission from Ref. [25] (copyright 2021, Xing). (**E**) Nanoparticle lysosome colocalization. Reprinted with permission from Ref. [25] (copyright 2021, Xing).

Although the nanoparticle drug delivery system has shown great advantages in oral administration, most current research is limited to their overall efficacy, and there is no systematic study on the transport and absorption mechanism of nanoparticles in the gastrointestinal tract [19]. Studying the transport mechanism of nanoparticles on the intestinal epithelial cell membrane is beneficial for guiding the design of carrier structures and improving the transport efficiency of drug-loaded nanoparticles across the membrane. Existing studies have shown that the nanoparticle transmembrane pathway can be roughly divided into three types, which are the paracellular pathway, the M cell pathway, and the enterocyte pathway [20]. Among them, intestinal epithelial cells are the most abundant cells in the intestine [21]. Clarifying the transport pathway of intestinal epithelial cells has important guiding significance for the transintestinal cell membrane of nanoparticles. As shown in Figure 1, this review focuses on the three processes involved in the transport of nanoparticles across the intestinal epithelium, which are cellular uptake, intracellular transport and basolateral exocytosis [22,23]. We hope to provide useful ideas and references for subsequent researchers in the design and synthesis of nanoparticle carrier structures.

## 2. Non-Intestinal Epithelial Cell Pathway

**Figure 2:**
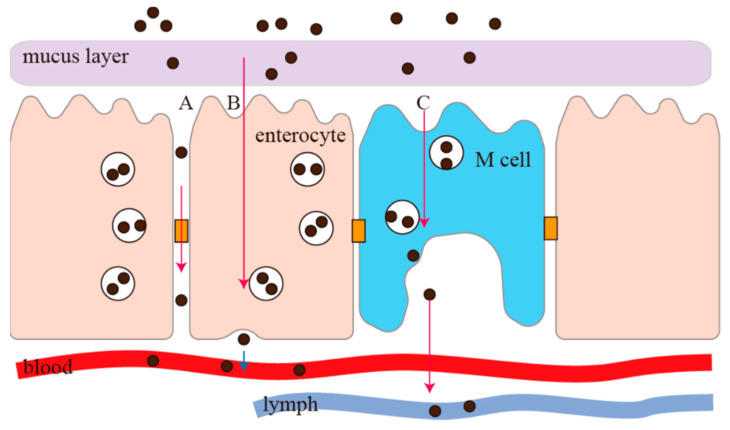
The pathway of nanoparticles across the intestinal cell membrane. A: Paracellular pathway; B: intestinal epithelial pathways; C: M cell pathway.

Most water-soluble macromolecular drugs and hydrophobic drugs have poor permeability, which restricts the oral bioavailability of these drugs [27,28,29]. Therefore, it is of great significance for the study of nanoparticles to deeply understand the absorption and transport mechanism of nanoparticles in the intestine. As shown in Figure 2, there are two pathways for nanoparticles to cross the membrane of gastrointestinal epithelium after entering the gastrointestinal tract, namely the non-intestinal epithelial and intestinal epithelial pathways. Non-intestinal epithelial cell pathways (transmembrane pathways that do not pass through intestinal epithelial cells) can be further divided into the paracellular pathway and M-cell pathway [30].

### 2.1. Paracellular Pathway

The paracellular route refers to the passage of nanoparticles through the intercellular spaces of the intestinal epithelial cell layer into the systemic circulation. However, there are tight junctions between cells in the intestinal epithelial cell layer. It has been reported that the allowable size range of tight connections under normal conditions is 0.3–1 nm [31]. Even if they are completely opened, the diameter of the open channel of tight junctions is no greater than 20 nm, which greatly restricts the permeability of the paracellular pathway [32]. Therefore, the ability of nanoparticles to cross the intestinal epithelial cell membrane via the paracellular pathway is severely restricted. In recent years, researchers have found that the tight junctions between intestinal epithelial cells are dynamic and are affected by actin contraction and intracellular calcium concentrations. For example, chitosan [33], polyacrylates [34] and thiopolymers can affect the Ca^2+^ complex in the tight junction structure via the interaction between the negative charge on the membrane and the positive charge on the polymer, thus opening the tight junction and improving the transcellular transport of nanoparticles [35,36]. The increase in intracellular Ca^2+^ can affect the phosphorylation of myosin, causing the contraction of light chains connecting the surrounding actin, opening tight junctions and enhancing the permeability of paracellular pathways [37]. Dou et al. [38] found that the uptake rate of chitosan-modified nanoparticles by Caco-2 cells was significantly higher than that of unmodified chitosan nanoparticles in a study of PLGA nanoparticles modified with chitosan. In addition, the uptake results showed a time and concentration dependence within a higher concentration range (25–100 μg/mL), and the uptake rate of modified nanoparticles was significantly higher than that of unmodified nanoparticles. However, the opening of tight junctions may increase the risk of autoimmune diseases, bacterial infections, and inflammatory bowel disease [39]. The safety of some penetration enhancers needs further consideration.

### 2.2. M Cell Pathway

M cells are activated phagocytes that are mainly distributed in the Peyer area of the intestinal tract, which can absorb antigens and microorganisms in the intestinal lumen and transport them to underlying lymphoid tissues [40]. Compared with ordinary intestinal epithelial cells, M cells have a unique structure. There are no dense brush cilia on the cell surface; only sparse villi structure and fewer mucous layers on the cell surface are observed, and the apical side of the cell is wrinkled and rich in vesicles. Therefore, the M cell pathway is an ideal pathway for exogenous substances to enter the systemic circulation. In addition, there are fewer M cell lysosomes than intestinal epithelial cells, and enzyme activity in the lysosome is lower [41], which reduces the degradation of nanoparticles in the process of intracellular transport and is conducive to the cross-cell transport of drugs encapsulated in nanoparticles [42]. Ma et al. [43] inserted Raji cells into Caco-2 cell monolayers to simulate the transport function of M cells, and the transcellular efficiency of nanoparticles loaded with silibinin–lipid conjugate was greatly improved. According to the different lipid chains, the efficiency of the transmembrane increased by 1.8-fold, 2-fold and 8-fold, indicating that the M cell pathway may be an important way to improve the transmembrane absorption of nanoparticles. However, a large number of studies have shown that M cells only account for 1% of the entire intestinal region [44]. Therefore, although the ability to ingest nanoparticles is very strong, the amount of transportation through M cells may also be limited. Secondly, the nanoparticles transported by M cells may be transported to the underlying lymphoid tissue and enter lymphatic circulation [45]. However, lymphatic circulation speed is only 1/500 of blood circulation speeds, and the individual difference is substantial. Therefore, the number of nanoparticles transported by the M cell pathway is limited, and drug-loaded nanoparticles must be transported through intestinal epithelial cells if they want to cross the intestinal epithelial cell membrane in large quantities.

## 3. Intestinal Cell Pathway

Intestinal epithelial cells are the most abundant epithelial cells in the intestine, accounting for 90–95% of intestinal cells [46]. They are columnar cells with hair-like projections called microvilli on the apical membrane, which greatly increase the surface area available for absorption [47]. The transintestinal cell pathway refers to the process in which nanoparticles pass through apical and basolateral membranes via intercellular transport and then discharge from the basement membrane to the extracellular space [48]. The entire process can be divided into three stages: the uptake of nanoparticles in the apical membrane of intestinal epithelial cells, the transport of endosomes in the cytoplasm and the exocytosis of nanoparticles in the basement membrane.

### 3.1. Endocytosis

**Figure 3:**
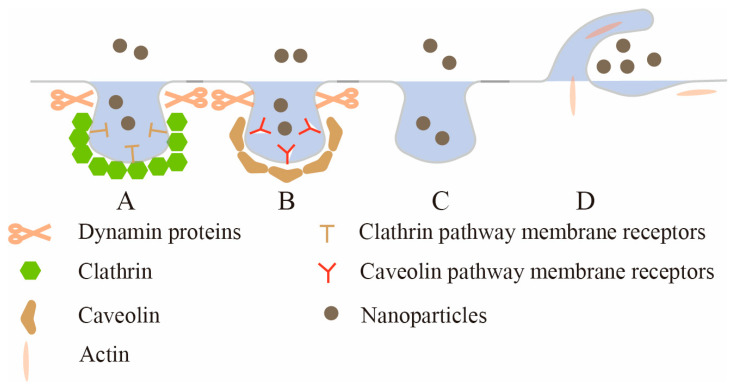
The uptake mechanism of intestinal epithelial cells. A: Clathrin-mediated endocytosis (CME); B: caveolae-mediated endocytosis (CavME); C: clathrin/caveolae-independent endocytosis (CIE); D: macropinocytosis.

Endocytosis is the process by which cells internalize proteins, lipids and other large molecules [49]. When nanoparticles reach the extracellular membrane, they can interact with the components of the plasma membrane or extracellular matrix, causing plasma membrane-coated nanoparticles to be entrapped in vesicles, which are subsequently pinched to form endocytic vesicles, and they are transported to specialized intracellular compartments [50]. Depending on the nanoparticle type and surface modification, there are several different routes of entry into the epithelium. In this article, according to the structural characteristics of intestinal epithelial cells, four endocytic pathways into epithelial cells are discussed. As shown in Figure 3, they are clathrin-mediated endocytosis (CME), caveolae-mediated endocytosis (CavME), clathrin/caveolae-independent endocytosis (CIE) and macropinocytosis.

Clathrin-mediated endocytosis (CME) is the major route for nanoparticles to enter cells, and many receptors on the epithelial membrane, such as transferrin receptor, low-density lipoprotein receptor, etc., can initiate CME [51]. Some investigators have previously referred to CME as receptor-mediated endocytosis, but this statement is not accurate because most pinocytic pathways also involve specific receptor–ligand interactions [52]. CME generally occurs in clathrin-rich plasma membrane regions, and the entire process can be divided into several stages: the initiation of clathrin-coated pits (CCP), cargo selection, CCP growth and maturation, scission and the release of clathrin-coated vesicles (CCV) [53]. CCP assembly is initiated by heterotetrameric adaptor protein 2 (AP2). First, abundant phosphatidylinositol lipid PI (4,5) P_2_ on the plasma membrane recruits the AP2 complex [54]; then, the AP2 complex rapidly recruits clathrin [55]. Clathrin has a three-legged structure comprising three heavy chains and three light chains, and this unique protein and other proteins (such as Eps15 and intersectin) spontaneously co-assemble into a complex structure that generates a curvature that stabilizes the membrane and enables vesicle budding: that is, CCP priming. As nascent pits grow, AP2 and other specific adaptor proteins recognize and recruit cargo, and adaptor and accessory proteins coordinate clathrin protein nucleation at the site of membrane internalization [56]. Clathrin nucleation would induce membrane invagination and stabilize the curvature of the pits. The Bin-Amphiphysin-Rvs (BAR) protein subsequently recruits the membrane scission protein dynamin, which aggregates to the neck of the budding vesicle. Finally, GTPase is hydrolyzed to drive membrane division [57], releasing the mature CCV from the plasma membrane. Huang et al. [58] modified TiO_2_ nanoparticles using transferrin and found that the nanoparticles would be taken up by cells through the CME pathway, and transferrin-modified TiO_2_ nanoparticles entered the cells faster than the unmodified TiO_2_ nanoparticles. In addition to surface ligand modification, some cellular factors such as epidermal growth factor (EGF) can also activate the CME process. Phuc et al. [59] found that the CME pathway was not involved in the uptake of polystyrene nanoparticles in the absence of EGF. However, in the presence of EGF, EGF was able to activate the CME pathway to enhance the cellular uptake of polystyrene nanoparticles.

Caveolae-mediated endocytosis (CavME) is another important pathway for receptor-specific nanoparticle internalization [60]. Unlike CME processes, which involve dynamic and sequential maturation, caveolae are 50–100 nm diameter bottle- or Ω-shaped pits located in the plasma membrane, where they exhibit a defined shape with consistent curvature and proportion in the neck region [61]. The outer surface of caveolae is usually covered with a layer of caveolin, a dimeric protein that binds to cholesterol, is inserted into the inner leaflet of the plasma membrane in a circular manner and binds to the surface of the membrane indentation to form a caveolin coating, which effectively stabilizes the bottle structure of caveolae [62]. Existing studies have shown that CavME is highly regulated and that caveolin stabilization at the plasma membrane is closely linked to actin stress fibers from Filamin A proteins that link caveolin to actin fibers and anchor caveolin to the plasma membrane. The germination of caveolae is regulated by kinase and phosphatase [63]. Studies have shown that kinase inhibitors can inhibit CavME, and phosphatase inhibitors can enhance CavME, which is caused by the loss of connection between caveolae and actin fibers after the rapid phosphorylation of Filamin A protein that is mediated by protein kinase Cα [64]. Once caveolae are detached from the plasma membrane, compared with vesicles produced by the CME pathway, vesicles produced by the CavME pathway are more likely to be transported to the Golgi apparatus and endoplasmic reticulum to avoid lysosomes and protect contents from the degradation of hydrolase in lysosomes [65]. Therefore, if intracellular or organellar targeting is desired, caveolae-mediated endocytosis is an exploitable route. For example, Cao et al. [66] prepared a kind of PEG nanoparticles and connected divalent folic acid to PEG. It was found that with the increase in divalent folic acid, the endocytosis pathway of nanoparticles changed from clathrin-mediated to a caveolin-mediated endocytosis pathway, and the localization of nanoparticles in lysosomes also decreased. Xin et al. [67] prepared a rod-shaped active pure drug nanoparticle, which was found to enter the cell using caveolae-mediated endocytosis, bypass the lysosome and enter the cytoplasm so that the drug is protected from lysosomal degradation. In summary, caveolae-mediated endocytosis can render nanoparticles prone to bypass lysosomes after entering cells and protect drugs from degradation by the action of hydrolytic enzymes in lysosomes. This makes it possible for more nanoparticles to be exocytosed out of the epithelium, reducing their retention inside the cell and, to some extent, increasing their transcellular efficiency.

Clathrin/caveolae-independent endocytosis (CIE), another important cell endocytic pathway, involves neither the formation of clathrin coats nor caveolae formation [68]. And endocytic vesicles involved in CIE have no obvious outer shell [69], so observing them using electron microscopy is difficult. This pathway was first discovered because nanoparticles were still found to be taken into cells after using inhibitors that block CME and CavME, indicating that other endocytosis pathways independent of CME and CavME exist in cells and such endocytosis pathways are resistant to CME and CavME inhibitors, namely CIE [70]. The CIE pathways reported so far can be classified according to whether dynamin proteins are used for membrane separation. RhoA (Ras Homolog Family Member A)-mediated endocytosis, fast endophilin-mediated endocytosis, Shiga-toxin-induced endocytosis, and ARF6 (ADP-ribosylation factor 6)-mediated endocytosis are all CIEs that require dynamin proteins for membrane separation. CDC42 (Cell Division Control protein 42)-dependent endocytosis and Flotillin-mediated endocytosis are CIEs that do not require dynamin proteins for membrane separation [71]. Among them, RhoA-mediated endocytosis, initially thought to be initiated by the activation of the interleukin 2 receptor, is also currently demonstrated to mediate the uptake of many cytokine receptors and their components [72]. ARF6-mediated endocytosis is currently demonstrated to activate phosphatidylinositol-4-phosphate-5-kinase by ARF6, generating phosphoinositide PI (4,5) P_2_, stimulating actin assembly and driving endocytosis [73]. CDC42-dependent endocytosis is a clathrin-independent and dynamin-independent pathway involving small GTPase enzymes Rac1 and CDC42, resulting in clathrin-independent carriers (CLICs). These CLICs fuse to form a special early internal compartment, called glycosylphosphatidylinositol-anchored protein-enriched internal compartments (GEECs), so this process is also known as the CLIC/GEEC pathway [74]. However, the degree of overlap between these pathways remains incompletely understood, and more molecular mechanistic studies are currently needed to address these questions and better define these pathways.

Macropinocytosis is a type of endocytosis in which cells nonspecifically take up extracellular fluid and extracellular macromolecules into large intracellular vesicles [75]. Some studies have shown that macropinocytosis is a process driven by actin, and the actin polymerization ring first forms protrusions on the plasma membrane, which are known as membrane folds. As the membrane fold continues to develop, the fold bends inwardly back to the plasma membrane to form a large vesicle [76]. The large vesicles formed in this process are also called macropinosomes, with a diameter of about 0.2–10 μm, which is significantly larger than that of RME (retromer-mediated endosome) [77]. Therefore, 70 KD glucan molecules are commonly used to mark macropinosomes with large sizes for studying macropinocytosis because these molecules are too large to enter cells via other endocytic mechanisms [78]. In addition, the entire endocytic process is regulated by a series of small GTPases, such as Ras GTPases that activate phosphatidylinositol 3-kinase to generate a membrane domain rich in phosphoinositol PIP_3_. These domains serve as docking sites for Rho GTPases, regulating actin remodeling and driving the formation of membrane ruffles [79]. After the formation of macropinosomes on the plasma membrane, Rab GTPases and phosphoinositides regulate a series of steps in their maturation. For example, Rab5 and Rab34 participate in the early stages of macropinosome formation, facilitating their fusion with early endosomes. After the maturation of the macropinosome, Rab7 replaces Rab5 to promote its fusion with late endosomes or lysosomes [80]. Nanoparticles tend to exhibit different uptake mechanisms depending on the material and particle size. As shown in Table 1, in general, nanoparticles larger than 200 nm tend to enter the cell by macropinocytosis.

### 3.2. Intracellular Transport

In the previous sections, different uptake pathways for nanoparticles have been discussed. After uptake, the next critical issue is the intracellular transport of nanoparticles, which plays a crucial role in determining their intracellular fate and their effect across the intestinal epithelial cell membrane. Most current studies focus on the surface modification of nanoparticles to enhance the uptake of nanoparticles. However, only some of the nanoparticles taken up by intestinal epithelial cells would be transported to the basal side, and others would either remain in the cell after internalization into the cell or are transported to lysosomes for degradation, resulting in fewer nanoparticles entering the blood across the epithelial membrane. This “easy uptake, difficult transport” problem for nanoparticles makes it necessary to elaborate on the pathways and regulatory mechanisms of the intracellular transport of nanoparticles across the intestinal epithelium. Therefore, this review summarizes the intracellular transport of nanoparticles across the intestinal epithelium from three aspects: endosomal circulatory system, enzyme regulation and intracellular trafficking pathways.

#### 3.2.1. Endosomal Circulatory System

After nanoparticles are internalized into cellular endosomes by various uptake pathways, their ultimate intracellular fate is usually determined by intracellular sorting and transport mechanisms, which mainly consist of an endosomal network with the Golgi apparatus, endoplasmic reticulum and lysosomes [102]. Among them, endosomes are initially generated from the plasma membrane, and after being regulated by various enzymes at different stages of development, they can fuse with the Golgi apparatus, endoplasmic reticulum or lysosomes. Endosomes are normally found in the cytoplasm of most human cells and can be classified into three main types: early endosomes, recycling endosomes, and late endosomes, which together form an intracellular endosome network [103]. In general, after leaving the plasma membrane, endosomes can fuse with early endosomes. The particles in the endosome will also become part of the early endosomes, which then serve as a transit station to guide the further trafficking of nanoparticles to different cellular destinations [104]. Some nanoparticles are transported by vesicles into recycling endosomes and finally to the plasma membrane. As early endosomes mature into late endosomes, the remaining nanoparticles are sent to lysosomes for degradation as late endosomes associate with lysosomes or can fuse with the plasma membrane to deliver nanoparticles out of the enterocyte [105].

Early endosomes (EEs) are the first intracellular compartment into which vesicles enter after nanoparticles are internalized. It is also a highly dynamic structure with a high propensity for vesicle fusion. In intestinal epithelial cells, EEs exhibit an extremely complex pleomorphic compartment. Various endocytic proteins and lipids co-construct a number of functionally different microdomains on EEs to deliver nanoparticles to different destinations. Ras-associated protein 5 (Rab5) is the most studied essential protein for EE biogenesis, which binds to different effector proteins on vesicles, leading to the maturation of vesicles into EEs. The overexpression of the active form of Rab5 promotes fusion between different EEs to form a giant EE. Similarly, the inhibition of all three different Rab5 isoforms (a, b and c) results in the complete inhibition of the endosome system in vivo [106]. In addition, the formation process of EEs is very dynamic, so observing the interaction of nanoparticles with this compartment is very difficult, but Rab5 mutants can form long-lived large EEs, allowing the visualization of the interaction of nanoparticles and EEs [107].

Recycling endosomes (REs) are important endosomes that maintain the balance of the plasma membrane and facilitate the flow of intracellular materials. Intracellular recycling is generally divided into two types [108]. One is the fast-recycling pathway; after nanoparticles reach the EEs, regulators such as Rab4 and Rab35 rapidly initiate the cell’s fast-cycling mechanism to deliver the nanoparticles via recycling endosomes to the apical-side membrane of intestinal epithelial cells [109]. The other type is the slow-recycling pathway, in which nanoparticles are transported from EEs to REs. Under the action of enzymes, REs are recycled with nanoparticles into various organelles, such as the endoplasmic reticulum, Golgi apparatus and lysosomes, and they are finally recycled back to the plasma membrane [110]. In addition, there is a balance between endocytosis and the recycling of cells, which maintains the balance and stability of the plasma membrane. For example, recycling not only maintains the balance of the flow of phospholipids and proteins on the plasma membrane but also circulates internalized receptors back to the plasma membrane so that they can repeatedly bind to ligands to promote the re-occurrence of endocytosis [111]. Typical examples include G-protein-coupled receptors, transferrin receptors, low-density lipoprotein receptors, GLUT4 (glucose uptake transporters), etc. [112].

Late endosomes (LEs) are polyvesicular endosomes containing multiple intracavicular vesicles (ILVs). ILVs are composed of four oligomeric proteins: ESCRT 0, I, II and III. During the maturation of EEs into LEs, the ESCRT complex causes the invagination of the outer membrane of the endosome and then is extruded into the lumen to form ILVs [113]. If the gene expression of the ESCRT complex is silenced by tyrosine kinase, ILV formation can be inhibited, and the monolayers of LEs can be observed using electron microscopy [114]. The surface of LEs contains 13 transmembrane proteins: Niemann Pick C-1 (NPC1). The steroid-responsive domain of NPC1 can interact with cholesterol and other lipids to promote their circulation in cells, and the number and size of LEs in NPC1-deficient cells increase [115]. In addition, Rab27a or Rab27b can transport LEs to the periphery of cells and make them into exosomes due to exocrine ILVs, which deliver contents out of cells [116]. In addition to fusing with the plasma membrane, LEs can also fuse with lysosomes to form endolysosomes that expose the contents to degrading enzymes [117].

#### 3.2.2. Enzyme Regulation of Endosomes

The endosomal circulation network is a highly dynamic system in which transport and transformation between endosomes occur constantly. Therefore, to ensure that the right goods arrive at the right place at the right time, this highly dynamic system must be tightly regulated and regulated. The Rab enzyme family of the small GTPase family plays an important role in ensuring this. Rab enzymes control many aspects of membrane transport, including early endosomal to late endosomal transformation, transport between various endosomes, the fusion of endosomes with organelles and plasma membranes, etc. [118]. Early endosomes can be labeled by Rab5, which plays an important role in the biogenesis of the endosomal system by regulating the fusion of small vesicles entering cells with early endosomes. In addition, its Rab5A isoform also plays a key catalytic role during the progression of early endosomes to late endosomes. Late endosomes can be marked by Rab7, which is directly or indirectly involved in the development of the endosome lysosome system, including the transition of early endosomes to late endosomes, the transport of late endosomes to lysosomes, lysosome biogenesis and the fusion of late endosomes with lysosomes [119], whereas Rab5-Rab7 turnover is a hallmark of the early endosome to late endosome turnover. In this process, Rab5 together with phosphatidylinositol 3-phosphate (PtdIns3P) recruits a complex containing Mon1/SAND1 and Ccz1/CCZ1 proteins into the early endosomes. This complex has two important roles: On the one hand, the Mon1/SAND1 protein inhibits its activity by displacing guanine nucleotide exchange factor rabex-5 from Rab5; Ccz1/CCZ1 on the other hand functions as a guanine nucleotide exchange factor in Rab7, leading to the activation of Rab7. The activation of Rab7 in turn recruits TBC2, a GTPase-activating protein that inactivates Rab5, further reducing Rab5 activity and leading to the dissociation of Rab5 effectors such as EETA [120]. Thereafter, Rab7 formally displaces Rab5, and early endosomes transition into late endosomes [121]. In addition to Rab5 and Rab7, at least 20 different Rab enzymes are involved in the endosomal maturation process, such as Rab7b, Rab6 and Rab9, which are involved in endosomal transport to the Golgi apparatus [122], and Rab10 may act upstream of Rab5 to promote the recycling of glycosyl phosphatidylinositol (GPI)-anchored proteins [123]. Many other Rab enzymes require further investigation to define their mechanism of action.

#### 3.2.3. Intracellular Trafficking Pathways

**Figure 4:**
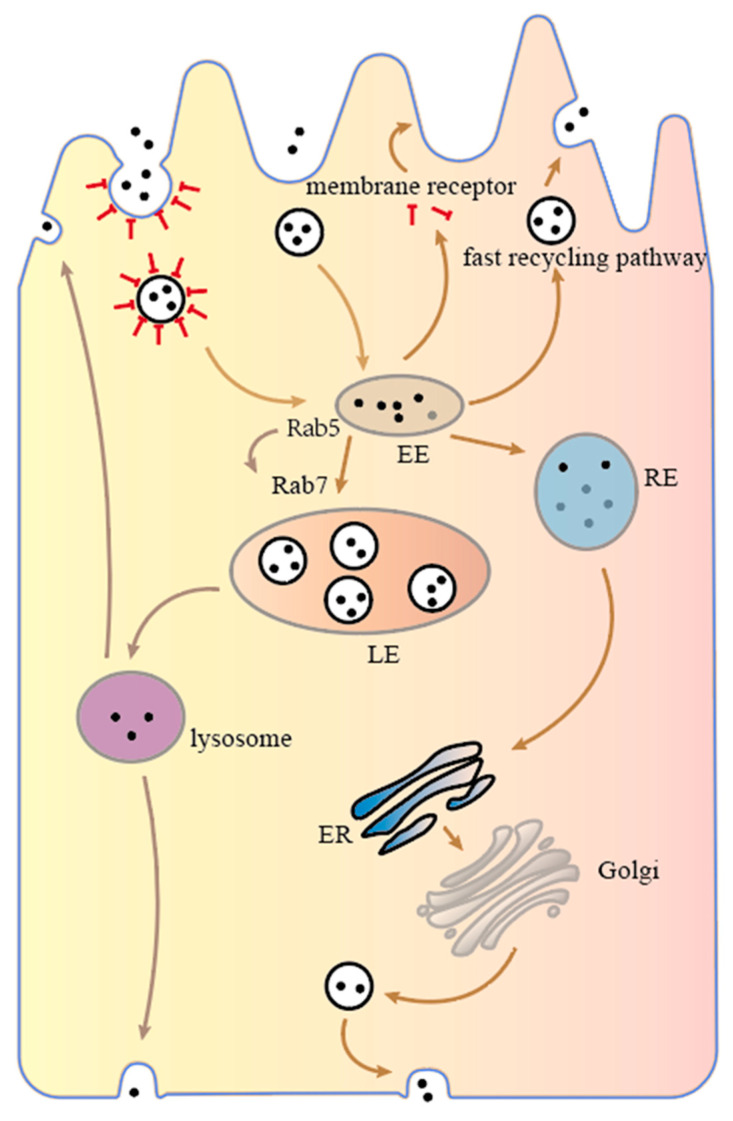
Intracellular transport mechanism of intestinal epithelial cells.

Based on the above review of the endosomal recycling network, we know that transport vesicles containing nanoparticles first fuse with the apical-side early endosome (EE), and then there are three pathways to transport nanoparticles according to the sorting transport mechanism within the cell [124]. As shown in Figure 4, (I) depending on the rapid recycling pathway, nanoparticles are sent back from the early endosome to the apical side of the intestinal epithelial cell. In addition, nanoparticles bound to internalized receptors can be released into the cytoplasm, where receptors return to the cell membrane in a rapid recycling pathway, and the nanoparticles continue to spread in the cytoplasm towards the basal side until they are degraded. (II) The nanoparticles are transported from early endosomes to late endosomes and eventually to lysosomes, which contain a wide range of hydrolytic enzymes capable of degrading almost all types of cellular components, such as proteins, fats, carbohydrates and even organelles. Nanoparticles that enter the lysosomes may be degraded or sent to both sides of the cell. (III) The nanoparticles are sent to recycling endosomes (REs), which then proceed to the ER, Golgi apparatus or late endosomes depending on the slow recycling pathway and eventually reach the basal side of the enterocyte where they are sent out of the cell, completing transcellular transport.

Based on the possible transport pathways of nanoparticles, we can summarize two important factors that need to be considered in the transcellular transport of nanoparticles. One is that when nanoparticles are transported from the apical side to the basal side, they are sent back to the apical side and then excreted back to the intestinal lumen, greatly reducing the transmembrane rate of nanoparticles [125]. The other is that the nanoparticles undergo degradation after they are transported to lysosomes during transport, which also greatly reduces the number of nanoparticles across the membrane [126]. Therefore, to improve transmembrane efficiency, inhibiting nanoparticles from refluxing to the apical side or being sent to lysosomes could be an important method.

### 3.3. Cellular Exocytosis

The ability of nanoparticles to enter cells does not equal the ability to cross cells. “Easy entry, difficult to cross cells” is a typical phenomenon in which cells cross intestinal epithelial cells [127]. The reasons for this phenomenon are numerous and complex. There are two possible reasons: First, internalized nanoparticles reflux to the apical side of intestinal epithelial cells, which are polarized cells, and the microstructure and proteins at the two ends of cells are not the same. Compared with the basal side, many nanoparticles have a greater tendency to egress from the apical side. The second is the intracellular degradation of nanoparticles. A typical example is the transport of nanoparticles to lysosomes, where they are degraded. Some studies have shown that the exocytosis rate of nanoparticles is slower than the internalization rate. Chai et al. [125], in their experiments studying solid lipid nanoparticles across Caco-2 cells, found that the exocytosis rate was only one-third of the internalization rate over a certain period. In general, exocytosis is even more difficult than internalization. Therefore, to improve the transcellular efficiency of nanoparticles, we need to pay more attention to exocytosis. However, unfortunately, current research studies on cellular exocytosis number far fewer than studies on cellular uptake. This paper focuses on two aspects of exocytosis combined with the analysis of the intracellular transport process of the upper segment. The two aspects are apical-side exocytosis and endoplasmic reticulum/Golgi exocytosis.

#### 3.3.1. Apical Exocytosis

The exocytosis of nanoparticles to the apical side is one of the important reasons affecting transcytosis efficiency. Although there is little research on this phenomenon, apical-side reflux can affect the transmembrane efficiency of nanoparticles. Some studies have reported that exocytosis on the apical side of nanoparticles is even greater than that on the basal side. For example, Wu et al. [128] modified polyethylene-glycol-coated nanoparticles using butyrate and found that the bilateral exocytosis of nanoparticles occurred, and significantly more nanoparticles were exocytosed from the apical side than from the basal side, which make up about 80% of the exocytosis from the apical side. Apical exocytosis greatly inhibits the transcellular effect of nanoparticles, which is not our ideal mode of exocytosis. More studies are needed to explore new strategies for apical exocytosis inhibition. Zhuang et al. [129] explored the effect of nanoparticle shape on their exocytosis on both sides of Caco-2 cells and found that rod-shaped nanoparticles not only had a greater tendency to enter cells than spherical nanoparticles but also had a greater tendency toward exocytosis on the basal side. In addition, Liu et al. [130] modified the surface of nanoparticles with angioproteinin-2 (Ang-2), which can target low-density lipoprotein receptor-associated protein 1 (LRP-1) expressed in the intestine, and Ang-2 nanoparticles were able to increase the expression of LRP-1 on the apical side of epithelial cells and further induce their redistribution to the basal side such that exocytosis on the apical side of nanoparticles decreased and exocytosis on the basal side increased, thus leading to increased transepithelial membrane trafficking. Overall, there are still very few studies on apical-side exocytosis at present, and more studies are needed to explore the involved mechanisms and influencing factors.

#### 3.3.2. Endoplasmic Reticulum/Golgi Exocytosis

The trans-endoplasmic Golgi network is an important pathway for the outward transport of substances from the cell, and the entire process involves a series of organelles, such as the endoplasmic reticulum (ER), Golgi apparatus, etc. Thus, this pathway of exocytosis is also called the ER/Golgi pathway. In a study on the transcellular mechanism of solid lipid nanoparticles with brefeldin A, Chai et al. [125] evoked the retrograde transport of Golgi enzymes and the retrograde transport of nanoparticles back to the ER to block the ER/Golgi pathway, and they used monensin to disrupt the Golgi apparatus and inhibit the transport of cargo from the Golgi apparatus to the plasma membrane. It was observed that the intracellular residence of nanoparticles increased, and exocytosis was inhibited, indicating that the ER/Golgi pathway plays an important role in the exocytosis of nanoparticles. In addition, the exocytosis of nanoparticles via the ER/Golgi pathway can circumvent lysosomes, decrease the intracellular degradation of nanoparticles and increase their rate of egress. Xing et al. [25] modified nanoparticles with L-cysteine, an amino acid related to Golgi localization. Studies on the exocytosis mechanism showed that the modification of cysteine enabled nanoparticles to be transported through the Golgi secretory pathway and bypass lysosomes. Thiol groups in cysteines have an important regulatory effect on Golgi transport, with cargo transported through the Golgi tending to be exocytosed out of the cell, avoiding lysosomal degradation. In addition to bypassing the lysosome, the ER/Golgi pathway is also indicated by its secretory function, Zhang et al. [26] modified two nanocarriers using a sorting signal peptide to direct the nanocarriers to the trans-Golgi (TGN) and basement membrane in order to compare the exocytotic efficacy of the two nanocarriers. Trans-Golgi-directed nanocarriers showed a significant advantage with respect to exocytosis, while basement-membrane-directed nanocarriers showed no significant difference from blank nanocarriers. The strategy of directing nanocarriers to the basement membrane only led to vesicle accumulation near the basement membrane. Therefore, the secretory function of the ER/Golgi pathway may have played an important role during exocytosis. This strategy, based on the physiological function of the trans-Golgi network to overcome the transcellular biological barriers of nanoparticles, has promising applications in polarized epithelial cells.

## 4. Conclusions and Outlook

Nanoparticle administration not only protects drugs from degradation in the physiological environment of the gastrointestinal tract but also facilitates the transport of drugs across the intestinal physiological barrier into the blood by various means of modifications. Intestinal epithelial cells are the main cells of the intestinal surface cell membrane, and a thorough understanding of the mechanism and strategy of nanoparticles crossing intestinal epithelial cells can not only help improve the bioavailability and blood concentration of drugs and increase the therapeutic effect but also reduces the administered dose based on increased transmembrane efficiency, alleviating adverse drug reactions and improving the treatment experience of patients. Therefore, this paper illustrates the transcytotic mechanism and strategy of nanoparticles from three aspects: the cellular uptake, intracellular transport and cellular exocytosis of nanoparticles in intestinal epithelial cells. Nanoparticles have the problem of being “ easy to access, difficult to cross cells “ inside the cell, and entry is not necessarily efficient. Methods for avoiding lysosomes and reducing apical exocytosis during intracellular trafficking need further investigation in order to explore the mechanism and propose more strategies. In addition, the ER/Golgi pathway appears to be an ideal exocytotic pathway that not only circumvents lysosomal degradation but also uses its secretory capacity to deliver nanoparticles out of the cell. It is anticipated that nanoparticle modification strategies based on this area will be another promising avenue toward nanoparticle modification. At present, researchers can design nanoparticles for different stages of transcellular migration, such as changing the material or particle size of nanoparticles to promote uptake, modifying the material to reduce lysosomal degradation during transport or promoting exocytosis. Increasing the absorption of nanoparticles to promote drug efficacy may be another design idea for nanoparticles. However, the integrity of nanoparticles after they cross the membrane and the mechanism of their drug release are currently not completely clear, and more studies are needed to gain insight into the specific changes in nanoparticles before and after they cross the cell in order to provide a reference and basis for the modification and design of nanocarriers.

## Figures and Tables

**Table 1:** Uptake mechanism of nanoparticles of different materials in epithelial cells.

Carrier Materials or Modification Materials	Particle Size	Cell Type	Cell Uptake Mechanism	Reference
polystyrene nanoparticles	100 nm	MDCK	CME	[81]
MPEG-PTMC diblock copolymer	127 nm	MDCK	CME	[82]
natural nanoparticles isolated from Coptidis	166 nm	MDCK	CavME	[83]
octaarginine-modified nanocarriers	200 nm	MDCK	CME, Macropinocytosis	[84]
Pr^3+^:LaF3 (CPr = 1%) nanoparticles	300 nm	MDCK	Macropinocytosis	[85]
transferrin-receptor-specific 7-peptide-modified nanoparticles	35 nm	Caco-2	CME	[86]
folic-acid-Pluronic-poly(lactide-co-glycolide) polymersome	76 nm	Caco-2	CME	[87]
thiolated nanostructured lipid carriers	190 nm	Caco-2	CME, CIE	[88]
PLGA nanoparticles	183 nm	Caco-2	CME	[89]
galactosylated albumin nanoparticles	116 nm	Caco-2	CME	[90]
vitamin B-12-modified trimethyl chitosan nanoparticles	321 nm	Caco-2	CME, CavME	[91]
stearic-acid-modified gelatin nanoparticles	247 nm	Caco-2	CME, CavME	[92]
apple-derived nanoparticle	170 nm	Caco-2	CME	[93]
soy protein nanoparticles	100 nm	Caco-2	CME, Macropinocytosis	[94]
barley protein nanoparticles	351 nm	Caco-2	CME, CavME	[95]
chitosan-modified PLGA nanoparticles	472 nm	Caco-2	CME, Macropinocytosis	[38]
TPGS-modified nanoparticles	114 nm	Caco-2	CME, CavME	[96]
oleoyl alginate ester nanoparticles	120 nm	Caco-2	CME	[97]
	420 nm	Caco-2	CavME	[97]
	730 nm	Caco-2	Macropinocytosis	[97]
zein pectin core/shell nanoparticle	253 nm	Caco-2	CME, CavME, Macropinocytosis	[98]
chitosan-modified nanoparticles	165 nm	Caco-2	CavME	[99]
chitosan-coated epigallocatechin-3-gallate-hordein nanoparticles	296 nm	Caco-2 /HT29	CavME, Macropinocytosis	[100]
hydroxypropyl beta-cyclodextrin-modified SLNs	187 nm	Caco-2	CavME, Macropinocytosis	[101]

Table abbreviations: CME denotes clathrin-mediated endocytosis; CavME denotes caveolae-mediated endocytosis; CIE denotes clathrin/caveolae-independent endocytosis.

## Data Availability

Data sharing does not apply to this article as no new data were created or analyzed in this study.
